# In Vitro Antibiofilm and Antibacterial Properties of Rue (*Ruta chalepensis*) and Garlic (*Allium sativum*) against Selected Bacterial Strains

**DOI:** 10.1155/ijm/9445940

**Published:** 2026-06-17

**Authors:** Fitsum Dejene, Ribka Getu, Dinka Ejeta, Yonas Syraji

**Affiliations:** ^1^ Department of Biology, College of Natural Sciences, Arba Minch University, Arba Minch, Ethiopia, amu.edu.et

**Keywords:** *Allium sativum*, antibiofilm, *Ruta chalepensis*, *Staphylococcus aureus*

## Abstract

This study is aimed at evaluating the phytochemical constituents and antimicrobial potential of *Ruta chalepensis* (Rue) and *Allium sativum* (Garlic), with a focus on their antibiofilm activity against selected bacterial strains. Ethanolic extracts of *R. chalepensis* leaves and fresh *A. sativum* bulbs were subjected to qualitative phytochemical screening, antibacterial testing, biofilm suppression assay, FT‐IR spectroscopy, and elemental analysis. Phytochemical analysis confirmed the presence of metabolites including phenols, tannins, saponins, alkaloids, and terpenoids. The extracts exhibited significant antibacterial activity (*p* < 0.05) against all tested reference strains. In the biofilm suppression assay using a microtiter plate method and crystal violet staining, both extracts effectively inhibited biofilm formation, with absorbance values ranging from 0.6–1.22 for *A. sativum* and 0.45–0.88 for *R. chalepensis*, compared to the untreated control. FT‐IR spectroscopy identified functional groups such as hydroxyl, carbonyl, carboxylic, and organosulfur compounds in garlic, and N‐H, C‐H aliphatic, C=C unsaturated, and aromatic rings in rue. Flame atomic absorption spectroscopy (FAAS) revealed essential elements including K, P, Mg, Ca, and Al in *A. sativum*, and Zn, Cu, Cr, and Mo in *R. chalepensis*. These findings support the traditional use of these plants in Ethiopian medicine and underscore their potential as natural, affordable alternatives for managing biofilm‐associated infections, particularly in oral health applications.

## 1. Introduction

Microorganisms do not exist solely as dispersed individual cells; instead, they aggregate at interfaces to form complex polymicrobial structures such as films, mats, flakes, sludge, or “biofilms” [1]. A microbial biofilm represents a specialized community of microorganisms adhering within an intricate extra polymeric matrix [2]. Typically, microorganisms constitute less than 10% of the dry mass in biofilms, with the matrix comprising more than 90%. The matrix, which is predominantly synthesized by the organisms themselves, serves as an extracellular material in which the biofilm cells are encapsulated. The biofilm structure is established through the intricate composition of extracellular polymeric substances (EPS), which encompass diverse biopolymers. These EPS components serve critical roles in the biofilm, including providing a scaffold for three‐dimensional architecture, facilitating adhesion to surfaces and promoting cohesion within the biofilm [3].

*Staphylococcus aureus*, a pathogen known for its ability to form biofilms, is implicated in chronic infections facilitated by its resistance to therapeutic interventions. This resilience is particularly evident in implanted medical devices like artificial heart valves, catheters, and joint prostheses [4, 5]. Biofilm related infections are known to cause significant health complications and increase the risk of morbidity and mortality. Medical device infections often require surgical removal and prolonged hospitalization, resulting in higher healthcare costs. Over the past decade, the prevalence of staphylococcal disease, including that caused by *S. aureus* has resulted in a significant increase in costs with an estimated annual cost of approximately $450 million [6, 7].

In regions such as Africa and many Asian countries, folk medicine is widely practiced leading to a widespread pursuit of herbal remedies. Traditional healers and their use of plant‐based medicines serve as the primary healthcare providers for a significant portion of the population in these developing nations. However, it is important to note that the focus of their healthcare approach is primarily curative rather than preventive addressing common ailments [8]. The accessibility and cost effectiveness of plants as direct therapeutic agents make them a compelling alternative to modern medicine. Consequently, there is now a substantial body of literature that focuses on the use of traditional medicine. Botanists provide descriptions of plants used for treating diseases, physiochemists investigate the chemical constituents of these plants, and pharmacologists assess the efficacy of specific plant compounds or extracts [9].

*Allium sativum*, commonly known as garlic, belongs to the Amaryllidaceae family. Garlic is native to Central Asia and has been consumed and used by humans for several thousand years. It has long been a widely used spice around the world [10]. On the other hand, *Ruta chalepensis*, commonly called “Tenadam” locally, is a group of small shrubs distributed in temperate and tropical countries and was introduced to America after the Spanish conquest. Different parts of this plant have been used in traditional medicine for centuries to treat various types of diseases [11].

*A. sativum* and *R. chalepensis* were selected for this study due to their documented bioactive properties and traditional medicinal use, particularly their potential antibiofilm activity. Although *S. aureus* is a well‐known biofilm‐forming pathogen, additional clinically relevant bacterial strains were included to evaluate the broader antimicrobial potential of these extracts. Although previous studies have investigated garlic extracts, including fresh juice, the current work addresses key gaps by systematically comparing the biofilm inhibitory effects of both extracts under controlled extraction conditions and by characterizing their chemical profiles, thereby providing novel insights into their antimicrobial and antibiofilm potential. Previous research concerning plants and their active constituents has primarily concentrated on their effects against free‐living bacteria, whereas biofilm forming bacteria known for their heightened resistance to antimicrobial agents have received relatively slight attention. This is significant as biofilms present greater challenges in terms of antimicrobial control strategies. This study is aimed at exploring the in vitro antibiofilm and antibacterial efficacy of rue (*R. chalepensis*) and garlic bulb (*A. sativum*) against selected bacterial strains.

## 2. Materials and Methods

### 2.1. Collection and Identification of Plant Materials

A laboratory‐based experimental study was conducted from June 4 to September 1, 2022, at the Microbiology and Parasitology Laboratory of Arba Minch University. The plant materials were collected with appropriate permission from local authorities, in accordance with institutional and national guidelines. The plant specimens were formally identified by Assistant Professor Mulugeta Kebebew—a botanist in the Department of Biology, College of Natural and Computational Sciences, Arba Minch University, Ethiopia—using standard taxonomic methods and reference materials. Voucher specimens have been deposited in the Arba Minch University Herbarium and are publicly accessible under the following accession numbers: AMU‐FRDY‐2025‐106 (*R. chalepensis)* and AMU‐FRDY‐2025‐107 (*A. sativum*). All experimental procedures were performed in triplicate. For the extraction of the antibiofilm agents, garlic bulbs (*A. sativum*) and rue (*R. chalepensis*) were collected. The samples were aseptically gathered using sterilized containers from various locations within Arba Minch town. Arba Minch is situated in the Southern Nations, Nationalities, and Peoples Region (SNNPR) and serves as the administrative center of the Gamo Zone. It is located 504 km from the capital, Addis Ababa, at 30°56 ^′^N latitude and 37°44 ^′^E longitude, covering an area of 2184 hectares, with an average temperature of 30.6°C and an annual rainfall of 575 mm [12]. All plant materials, except for the garlic bulbs, were dried in the shade until all moisture had evaporated, ensuring they were adequately prepared for grinding. After drying, the plant materials were finely ground using a mechanical blender and transferred to airtight containers, which were properly labeled for future use.

### 2.2. Preparing the Garlic Extract

Fresh ripened garlic bulbs were purchased from Arba Minch city market. The cleaned garlic bulbs were peeled, weighed (20 g), and aseptically homogenized using a sterile flour mill (IKA A11 BASIC, D‐79219 Staufen). The homogenized garlic was then filtered through sterile cheesecloth. The filtrate was stored in 15 mL test tubes at 20°C until use. From 20 g of raw garlic, 3 g of raw garlic extract was obtained. Subsequently, a stock solution of 300 mg/mL was prepared using a 10% DMSO solution (10 mL), which was considered to be at a concentration of 100% [13, 14].

### 2.3. Preparing the Rue Extract

The Rue plants (*R. chalepensis*) were collected at various stages of growth from Arba Minch district. The test leaves were dried in the shade at room temperature and then ground into a fine powder using an electric flour mill (IKA A11 BASIC, D‐79219 Staufen). Extraction with ethanol and water was performed according to established procedures [15]. Twenty grams of the individual powdered leaves were homogenized in 200 mL of 97% ethanol and distilled water in 250 mL Erlenmeyer flasks. The samples were shaken at room temperature at 140 rpm for 24 h. The coarse materials were removed by centrifugation at 3000 rpm for 5 min. The supernatant was filtered using Whatman filter paper No. 1 (DP 1507110‐Batch HJ7621) and then concentrated to dryness at a water bath temperature of 40°C using a rotary evaporator (F‐MODEL‐RSE‐065 L.NO‐LAP‐137). The dried materials were subsequently dissolved in 10 mL of distilled water.

### 2.4. Qualitative Determination of Phytochemical Constituents

In separate conical flasks, 5 g of samples were soaked for 72 h in methanol and distilled water. A phytochemical screening method was used to identify and evaluate the presence of secondary metabolites, including steroids, flavonoids, saponins, alkaloids, phenols, tannins, and terpenoids. The extracts were filtered. Then for the crude extracts of methanol and distilled water, phytochemical screening was conducted [16, 17].

#### 2.4.1. Test for Steroids

A mixture of 1 mL of methanolic extract and 1 mL of chloroform, along with 2–3 mL of acetic anhydride and 1–2 drops of concentrated H_2_SO_4_, was prepared. When three drops of concentrated H_2_SO_4_ were added to 3 mL of the extract and a red coloration was observed, this indicated the presence of steroids [17, 18].

#### 2.4.2. Test for Flavonoids

One milliliter of 10% lead acetate was added to 1 mL of the extract contained in a test tube. The formation of a yellow precipitate was considered a positive indicator for flavonoids [18].

#### 2.4.3. Test of Saponins

Three milliliter of the extract was mixed with 5 mL of distilled water in a test tube and shaken vigorously for 2 min. The formation of stable foam indicated the presence of saponins [18].

#### 2.4.4. Test for Alkaloids

Two milliliter of aqueous extract in a test tube was mixed with two drops of 1.5% HCl and then filtered. The resulting 2 mL of filtrate from the plant drug extract was combined with 2 mL of Wagner′s reagent. The formation of a reddish‐brown precipitate indicated the presence of alkaloids [16, 18].

#### 2.4.5. Test of Phenols

One milliliter of the aqueous extract was mixed with 3–4 drops of 5% FeCl_3_ (w/v). The formation of a bluish‐black color indicates the presence of phenols [18].

#### 2.4.6. Test for Tannins

Two milliliter of extract was placed in a test tube, and 3–4 drops of 5% ferric chloride solution were added. The formation of a dark blue color indicates the presence of tannins [16, 18].

#### 2.4.7. Test for Terpenoids

A test for terpenoids was conducted by mixing 3 mL of extract with 2 mL of chloroform in a test tube. Subsequently, 2 mL of concentrated H_2_SO_4_ was added gently to form a ring layer that interfaced with a reddish‐brown color, indicating the presence of terpenoids [17, 18].

### 2.5. Standard Strains

The bacterial strains utilized in this study were *S. aureus* ATCC 25923, *Escherichia coli* ATCC 25922, and *Salmonella gallinarum* ATCC 9184. These strains were obtained from the Ethiopian Public Health Institute (EPHI) collection center. Following their growth on mannitol salt agar, a semisynthetic medium designed for the selective cultivation of bacteria, the strains were sub cultured.

### 2.6. Antibacterial Activity

A 0.5 McFarland standard was prepared as described in reference [19]. The pathogenic bacterial strain was cultured on Mueller–Hinton (MH) agar and incubated for 24 h. Subsequently, 2–3 colonies were aseptically transferred into sterile saline using a wire loop, adjusting the turbidity to the 0.5 McFarland standard (equivalent to a concentration of 1.5 × 10^8^ CFU/mL). Each MH agar plate was uniformly inoculated with the bacterial suspension using a sterile swab, allowing excess liquid to be absorbed by the agar surface. Wells were created in the agar using a sterile cork borer (6 mm diameter). Sample solutions containing 140 mg/mL of *R. chalepensis* and 110 mg/mL of *A. sativum* were added to the wells using a micropipette. The plates were then incubated at 37°C for 24 h. The diameters of the inhibition zones were measured in millimeters, and the results were recorded accordingly. Zones with diameters less than 12 mm were considered to lack significant antibacterial activity. A positive control well contained chloramphenicol (32 *μ*g/mL), whereas sterile DMSO served as the negative control [20].

### 2.7. Biofilm Suppression Assay

Microbial biofilms were cultured in a 96‐well microtiter plate. Overnight cultures of *S. aureus* were diluted 1:100 in BHI broth, and 100 *μ*L of this diluted suspension was added to sterile, flat‐bottomed polystyrene 96‐well microtiter plates. After 24 h of incubation, the broth was carefully aspirated using a multichannel pipette. The wells were then rinsed three times with 100 *μ*L of phosphate‐buffered saline (PBS). Once the plates were completely dry, the biofilms were exposed to ethanolic extracts of *R. chalepensis* (at concentrations of 110, 33, 9.9, and 2.97 mg/mL) and aqueous extracts of *A. sativum* (at concentrations of 140, 42, 12.6, and 3.7 mg/mL) for 20 min. Subsequently, the extracts were carefully removed by pipetting, and the wells were washed with distilled water. The microtiter plates were inverted onto a blotting sheet and air‐dried. Biofilms were stained with 100 *μ*L of 0.1% crystal violet stain for 15 min, followed by washing with distilled water and air‐drying of the plates. To quantify the biofilms, 100 *μ*L of 30% acetic acid was added to each well to dissolve the biofilm. The optical density of the solubilized crystal violet stain was measured at 570 nm using an ELISA reader [2]. This assay evaluated biofilm inhibition, in which the test compounds were added during the initial bacterial inoculation to prevent biofilm formation.

### 2.8. Fourier Transform Infrared Spectroscopy (FT‐IR) Analysis

The FT‐IR is an exceptional instrument for identifying various functional groups in compounds. To capture the infrared absorption region, the FT‐IR spectrum was utilized to analyze different functional groups of the compounds based on their peak values within the IR spectra. According to Bobby et al. [21], the ground leaves of *R. chalepensis* and *A. sativum* were subjected to FT‐IR analysis, and the functional groups of the compounds were categorized based on the peak values of the dried powder of *R. chalepensis* used in the FT‐IR study. A total of 10 mg of the dried plant leaf extract powder was compressed with 100 mg of potassium bromide (KBr) to create translucent sample disks. The scanning range for the samples extended from 500 to 4000 cm^−1^ with a resolution of 1 cm^−1^.

### 2.9. Flame Atomic Absorption Spectroscopy (FAAS) Analysis

An amount of 0.5 g of air‐dried and homogenized samples of *R. chalepensis* and *A. sativum* were transferred to a 250 mL round‐bottom flask. Next, 3.5 mL of a mixture of nitric acid (69%–72%) and perchloric acid (70%) was added. The mixture was digested in a micro‐Kjeldahl digester at temperatures of 210°C and 230°C for durations of 1 h and 45 min and 2 h and 30 min, respectively. After digestion, the solution was allowed to cool for 20 min without removing the condenser, followed by an additional 10 min after its removal. Subsequently, 25 mL of distilled water was added to dissolve any precipitate formed during cooling and to minimize the dissolution of filter paper by the digested residue when filtered through Whatman filter paper. The round‐bottom flask was rinsed with 5 mL of distilled water, bringing the total volume to 45 mL. To this solution, a 1% lanthanum nitrate solution was added, and the volume was adjusted to 50 mL with distilled water. The digested samples were refrigerated until the analysis of metal content in the sample solutions was conducted using FAAS [22].

### 2.10. Data Quality Assurance

Data integrity was maintained throughout the entire process, from data acquisition to strain sub culturing, in accordance with established standard operating procedures (SOPs). The effectiveness of the prepared media was assessed by inoculating control strains, specifically *S. aureus*, *E. coli*, and *S. gallinarum* obtained from the EPHI. The culture medium was prepared according to the manufacturer′s instructions, and its sterility was confirmed by incubating 5% of the medium overnight at 35°C–37°C and inspecting for any bacterial growth. Media batches that exhibited growth were discarded and re‐prepared. To ensure data quality control for the experimental unit, both positive and negative control units were utilized.

### 2.11. Statistical Analysis

All experiments were conducted in triplicate. The data were analyzed using one‐way ANOVA, followed by post hoc Tukey tests performed with SPSS Version 25 (IBM Statistics, Armonk, New York, United States). A *p* value of 0.05 or lower was established as the threshold for statistical significance.

## 3. Results and Discussions

### 3.1. Phytochemical Analysis

Phytochemical screening revealed that both the powdered form of *R. chalepensis* and fresh juice of *A. sativum* contain the following chemical compounds (Table 1).

**Table 1:** Summary of phytochemical compounds in plant extracts.

Phytochemical	*Allium sativum*	*Ruta chalepensis* (^a^)	*Ruta chalepensis* (^b^)
Phenols	+	+	−
Tannins	+	+	−
Flavonoids	+	−	+
Saponins	+	+	+
Steroids	+	−	+
Alkaloids	+	+	+
Terpenoids	+	+	+

*Note:* “+” stands for the presence and “−” stands for the absence of the chemical constitutes.

^a^Ethanol crude extract and ^b^distill water crude extract.

Qualitative analyzes of ethanol and distilled water extracts of *R. chalepensis* and fresh juice of *A. sativum* were performed and several bioactive components were identified. Preliminary screening of phytochemicals in various extracts of *R. chalepensis* leaves and *A. sativum* stems was determined according to the standard protocol. Phytochemical screening of *A. sativum* extract showed the presence of terpenoids, alkaloids, phenol, tannin, flavonoids, and other components as shown in Table 1. On the other hand, phytochemical screening of the ethanolic extract of *R. chalepensis* showed the presence of phenol, tannins, saponins, alkaloids, and terpenoids. However, flavonoids and steroids were missing in the plant extract. Contrary to the above statement, phytochemical study of the distilled water extract of *R. chalepensis* revealed the presence of saponins, alkaloids and terpenoids, flavonoids, and other components except the bioactive component′s phenols/tannins.

### 3.2. Antibacterial Activities

The antibacterial activity of both plant extracts was tested against pathogenic bacteria and the presence or absence of inhibition zones on the agar well plates was monitored. In this test, three strains of pathogenic bacteria, *S. aureus*, *E. coli*, and *S. gallinarum*, were used to evaluate the antibacterial effects of *A. sativum* and *R. chalepensis* crude extract. As shown in Tables 2, 3, and 4, the crude extract of both medicinal plants showed strong inhibitory effects against all pathogenic bacteria tested. The majority of the extract exerted consistent antibacterial effects against *S. aureus*, *E. coli*, and *S. gallinarium*, with an inhibition zone diameter of 17–25 mm for *A. sativum* and 13–19 mm for *R. chalepensis*. The negative control of DMSO used in the study showed no inhibitory effect.

**Table 2:** Mean (in millimeter) zone of inhibition of *Allium sativum* extract for the tested bacteria.

No.	Concentration (mg/mL)	*E. coli*	*S. aureus*	*S. gallinarium*
1.	110	23 ± 1.00	25 ± 6.08	23 ± 2.91
2.	33	22 ± 2.00	24 ± 2.00	20 ± 1.00
3.	9.9	17 ± 3.61	19 ± 1.50	19 ± 1.32
4.	Cipro (5 mg)	23 ± 2.64	24 ± 1.64	25 ± 3.60

*Note:* Values are expressed as mean ± standard deviation (*n* = 4). Analysis was performed using a one‐way ANOVA followed by a Tukey test with post hoc multiple comparisons. As compared to the positive control—to 110, 33, and 9.9 mg/mL—the negative control, DMSO, showed no antibacterial activity.

Abbreviations: Cipro, ciprofloxacin as positive control; SD, standard deviation.

**Table 3:** Mean zone of inhibition (mm) of ethanolic extract of *Ruta chalepensis* against the tested bacteria

No.	Concentration (mg/mL)	*E. coli*	*S. aureus*	*S. gallinarium*
1.	140	18 ± 2.29	19 ± 1.32	17 ± 0.91
2.	42	16 ± 0.50	16 ± 1.80	15 ± 1.20
3.	12.6	14 ± 2.00	15 ± 0.86	13 ± 1.32
4.	Cipro (5 mg)	25 ± 3.60	23 ± 3.74	22 ± 1.00

*Notes:* Values are expressed as mean ± standard deviation (*n* = 4). Analysis was performed using one‐way ANOVA followed by a Tukey test with post hoc multiple comparisons. As compared to the positive control—to 140, 42, 12.6 mg/mL—the negative control, DMSO, showed no antibacterial activity.

Abbreviations: Cipro, ciprofloxacin as positive control; SD, standard deviation.

**Table 4:** Mean (in millimeter) zone of inhibition of distill water extract of *Ruta chalepensis* for tested bacteria.

No.	Concentration (mg/mL)	*E. coli*	*S. aureus*	*S. gallinarium*
1.	180	16 ± 0.86	19 ± 2.26	17 ± 3.60
2.	54	17 ± 1.80	16 ± 0.86	15 ± 1.73
3.	16.2	15 ± 1.20	14 ± 1.30	14 ± 1.00
4.	Cipro (5 mg)	25 ± 3.60	23 ± 2.91	22 ± 0.51

*Note:* Values are expressed as mean ± standard deviation (n = 4). Analysis was performed using a one‐way ANOVA followed by a Tukey test with post hoc multiple comparisons. As compared to the positive control—to 180, 54, 16.2 mg/mL—the negative control, DMSO, showed no antibacterial activity.

Abbreviations: Cipro, ciprofloxacin as positive control; SD, standard deviation.

When comparing the inhibition zones produced by the *A. sativum* plant extract of different concentrations, there was a statistically significant difference between the groups as shown by one‐way ANOVA (*F* (3, 8) = 5.687, *p* = 0.022). A Tukey post hoc test showed that there was no statistically significant difference between groups. However, there is a statistically significant difference for the concentration of 9.9 mg/mL compared to the concentration of 110 mg/mL of *A. sativum* (*p* < 0.05). Based on the inhibition zones created by *A. sativum* extract and the controls, the different concentrations of *A. sativum* were found to have inhibitory effects against the test pathogenic strains in the following descending order: 110 > 33 > 9.9 mg/mL. Among the test strains, the pronounced inhibition results in *S. aureus* were observed at 110 and 33 mg/mL, whereas 9.9 mg/mL showed the lowest inhibitory effect in all cases.

When comparing the inhibition zones produced by the ethanolic extract of *R. chalepensis* belonging to different concentrations, there was a statistically significant difference between the groups as shown by one‐way ANOVA (*F* (3, 8) = 37.194, *p* = 0.000). Based on the outcomes of the Tukey post hoc test, no statistically significant differences were observed between groups overall. However, significant statistical disparities were identified among all concentrations compared to the positive control and specifically between the concentrations of 140 and 12.6 mg/mL (*p* < 0.05). The effectiveness of the ethanolic plant extracts in inhibiting bacterial growth varied with the concentration used as indicated by the mean zone of inhibition. At 140 mg/mL, S. *gallinarum* exhibited the smallest inhibition zone diameter while other bacterial strains demonstrated substantial inhibition. Conversely, the concentration of 42 mg/mL displayed the most effective inhibition against *E. coli* and *S. aureus* with S. *gallinarum* showing the least inhibition. All tested bacteria exhibited significantly different inhibition zones compared to the positive control (ciprofloxacin, 5 mg/disc) across all concentrations of the plant extract (*p* < 0.05). In summary, the antibacterial efficacy of the plant extract correlated directly with its concentration.

When comparing the zones of inhibition produced by the distilled water extract of *R. chalepensis* belonging to different concentrations, there was a statistically significant difference between the groups as shown by one‐way ANOVA (*F* (3, 8) = 43.564, *p* < 0.001). A Tukey post hoc test showed that there was no statistically significant difference between groups, except for 180 mg/mL, which is significant compared to 16.2 mg/mL. However, compared to the positive control, there is a statistically significant difference for all concentrations. In general, the mean inhibition zones of the antibacterial activity of the distill water extract were dependent on its concentrations, as shown in Table 4.

In the in vitro antibacterial investigation, three strains of pathogenic bacteria, *S. aureus, E. coli*, and *S. gallinarium* were used to evaluate the antimicrobial effects of *A. sativum* and *R. chalepensis* crude extract. As confirmed by inhibitory zones in the agar well diffusion assay *A. sativum* was found to have inhibitory activity against the test pathogenic strains in the following descending order: 110 > 33 > 9.9 mg/mL. The result of this study can be corroborated with the previous reports on *A. sativum* that have demonstrated antibacterial effects against human pathogenic organisms [23]. On the other hand, *R. chalepensis* crude extract have also shown inhibitory activity against the test pathogenic strains in the following ascending order: Ethanol extract, 12.6 <42 <140 mg/mL and distill water, 180 <54 <16.2 mg/mL. A recent report by Alsaid et al. [23], Ohara et al.[24], and other author similarly demonstrated that crude extract of *A. sativum* and *R. chalepensis*, respectively, exert antimicrobial effect in vitro against the pathogenic organism [25].

The antibacterial activity of *A. sativum* and *R. chalepensis* crude extract was mainly investigated in vitro and the studies particularly focused on the inhibitory activity against the growth of gram negative and gram‐positive pathogens. An antibacterial substance of this crude extract of *A. sativum* and *R. chalepensis* strain exerts an inhibitory effect against *S. aureus*, *E*. *coli,* and *S. gallinarium* by producing antibacterial metabolites such as allicin in *A. sativum* and alkaloids, flavonoids, and tannin in *R. chalepensis* [14].

The antibacterial and antibiofilm effects reported in this study were observed at relatively high concentrations, exceeding levels typically considered pharmacologically feasible for systemic antimicrobial therapy. These findings should be interpreted as preliminary in vitro observations rather than as evidence supporting immediate clinical or drug development applicability. Future investigations should include quantitative MIC and MBC analyses, as well as cytotoxicity and formulation studies, to more rigorously evaluate the practical significance of the observed antimicrobial and antibiofilm activities.

### 3.3. Biofilm Inhibition Assay

**Figure 1:**
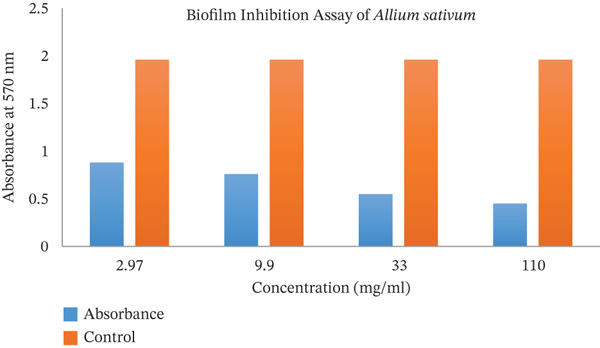
Absorbance values of crystal violet assay with single‐species biofilms produced by *S. aureus.*

**Figure 2:**
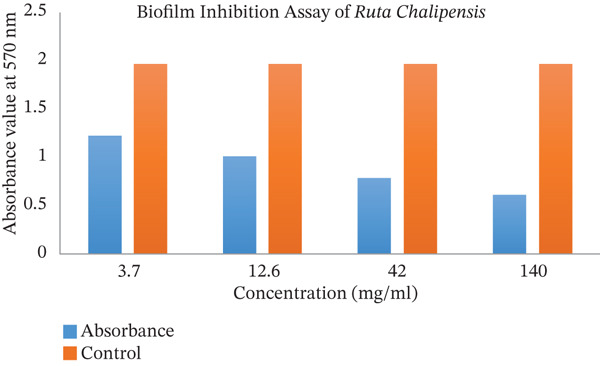
Absorbance values of crystal violet assay with single‐species biofilms produced by *S. aureus.*

The biofilm inhibition and activities of *A. sativum* and *R. chalepensis* extracts as individual agents on mature biofilms of a single species of *S. aureus* are shown in Figures 1 and 2, respectively.

Regarding the effects on the biofilm formation, the ethanolic extracts of *R. chalepensis* leaves and fresh *A. sativum* extract resulted in good biofilm inhibition of *S. aureus* biomass compared to the untreated control, as shown in Figures 1 and 2. In detail, both *A. sativum* and *R. chalepensis* extracts caused a significant decrease in the absorbance value at 570 nm wavelength for the *S. aureus* isolate in the range of (0.60–1.22) and (0.45–0.88), respectively, negative control with 1.96. It was found that the conducted biofilms of the tested isolate were significantly destroyed at different concentrations as shown in (Figures 1 and 2).

Biofilm inhibition assays show that *A. sativum* and *R. chalepensis* can inhibit *S. aureus* biofilm formation. Comparable results on the biofilm inhibition test of chamomile oil on the growth of the bacterial pathogens *Aggregatibacter actinomycetemcomitas* and *Treponema denticola* were previously reported by Nurrahman and Widyarman [26]. Similarly, Hans [27] and her colleague proved that chamomile essential oil effectively inhibits the growth of *S. aureus* and *P. gingivalis*. Chamomile may be associated with and exerts an antibacterial effect by disrupting membrane integrity and inhibiting cellular respiration. This could potentially involve ɑ‐bisabolol promoting the destruction of bacterial cell membranes thereby enabling their penetration by exogenous solutes. Therefore, we simply assumed that the same biological mechanism occurred when the plant test solution was added to wells containing *S. aureus*.

### 3.4. FT‐IR Result

**Figure 3:**
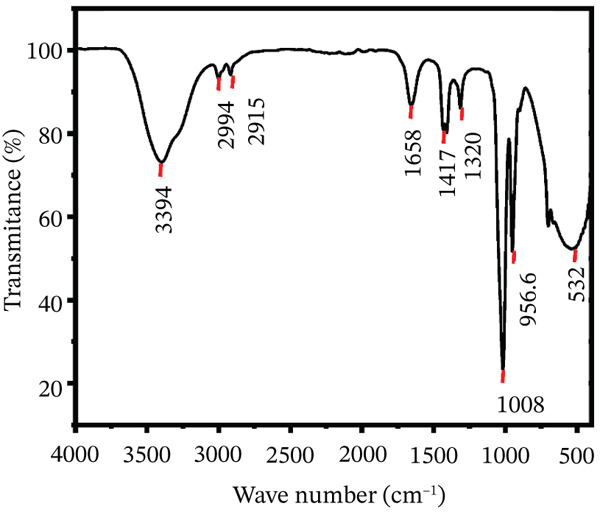
FT‐IR spectrum of extract of *Ruta chalepensis* leaf.

Table 5 and Figure 3 show the peak values and likely functional groups identified through FT‐IR analysis of the extracts. FT‐IR spectroscopy provides information on the predominant functional groups present, serving as a tool for chemical characterization of the extracts rather than identification of specific bioactive components.

**Table 5:** FT‐IR spectral wavenumber′s values and functional groups obtained from an extract of *Ruta chalepensis* leaf.

No	Peak value	Bond	Functional group
1	3394 cm^−1^	N–H	Nitrogen group
2	2994 cm^−1^	C=C	Unsaturated alkenes
3	2915 cm^−1^	C–H	Aliphatic group
4	1658 cm^−1^	C=C	Unsaturated alkenes
5	1417 cm^−1^	C–H	Stretching aliphatic
6	1320 cm^−1^	C=O	Carbonyl GROUP
7	1008 cm^−1^	C–O	C–O…group
8	956.6 cm^−1^	C=C	Trans unsaturated alkene
9	700 cm^−1^	Ring	Ring aromatic
10	532 cm^−1^	C–I	Halogen

The FT‐IR spectrum of *R. chalepensis* extracts showed peaks corresponding to functional groups such as nitrogen‐containing groups, unsaturated alkenes, aliphatic chains, aromatic rings, and trans‐unsaturated alkenes. These data provide information on the chemical composition of the extract but do not allow identification of individual bioactive compounds.

**Figure 4:**
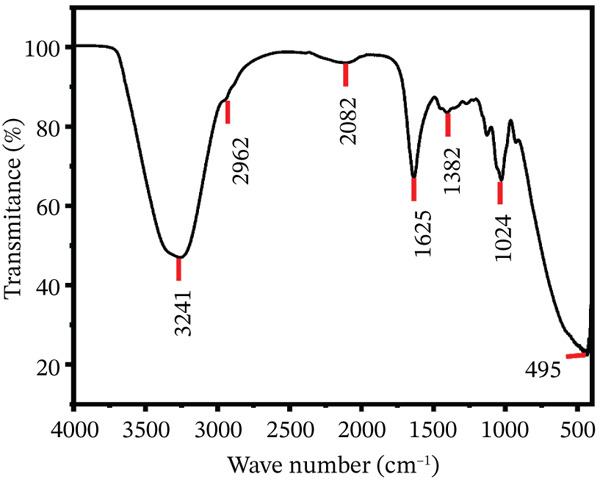
FT‐IR spectrum of aqoues extract of *Allium sativum*.

Conversely, FT‐IR analysis of the aqueous extract of *A. sativum* indicated peaks corresponding to functional groups such as carboxylic, hydroxyl, carbonyl, and organosulfur groups (Table 6 and Figure 4). These data provide information on the chemical composition of the extract, without identifying specific bioactive compounds.

**Table 6:** FT‐IR spectral wavenumber’s values and functional groups obtained from Aqoues extract of *Allium sativum*.

No	Peak value	Bond	Functional group
1	3241 cm^−1^	O–H	Hydroxyl group
2	2962 cm^−1^	C–H	Aromatic compound
3	2082 cm^−1^	C≡C	Alkynes
4	1625 cm^−1^	C=O	Carbonyl and carboxyl group
5	1382 cm^−1^	O–H	Carboxylic acids
6	1024 cm^−1^	S=O	Organosulfur compounds
7	495 cm^−1^	S–S	S–S stretching

The FT‐IR spectrum was used to characterize the functional groups present in the extracts based on the observed peak values in the IR region. The IR absorption band spectrum of *R. chalepensis* leaf extracts showed peaks corresponding to N–H, C–H stretching aliphatics, C–O groups, C=C unsaturated alkenes, trans‐unsaturated alkenes, and aromatic rings. Peak variations observed in the ethanolic extracts likely reflect differences in chemical composition and absorption patterns. GC‐MS analysis of *R. chalepensis* by Ayana and colleagues using different solvents reported the presence of ketones, alkenes, alcohols, and other functional groups in ethanolic and methanolic extracts [28]. In addition, Jaradat and colleagues reported that *R. chalepensis* essential oil has potential antifungal and antibacterial effects [29]. Although these studies indicate the biological relevance of the plant, the present FT‐IR data should be interpreted strictly as chemical characterization and do not provide direct evidence of antibacterial or antibiofilm mechanisms. The differences between our results and previous studies may be due to environmental and geographical factors, such as soil composition and climatic conditions, which can influence the chemical composition of plant extracts [30–32].

On the other hand, FT‐IR analysis of the aqueous extract of *A. sativum* revealed peaks corresponding to functional groups such as hydroxyl, carbonyl, carboxylic acid, and organosulfur‐related groups. The broad peak at 3241 cm^−1^ corresponds to O–H stretching vibrations, the peak at 2962 cm^−1^ to asymmetric C–H stretching of aromatic compounds, 1619 cm^−1^ to C=O stretching of carbonyl and carboxyl groups, 1382 cm^−1^ to O–H bending of carboxylic acids, and 1036 cm^−1^ to S=O stretching, consistent with organosulfur compounds. These FT‐IR findings are in agreement with previous reports by Songsungkan and Chanthai [33], which also showed hydroxyl, carbonyl, carboxyl, and organosulfur functional groups in methanolic *A. sativum* extracts [34]. It should be noted that FT‐IR analysis provides information on the presence of functional groups and does not allow identification of specific metabolites or direct inference of biological activity.

### 3.5. FAAS Result

The elemental analysis results obtained using the comparative method of FAAS techniques are presented in Table 7 in ppm weights of the samples. The results show that *R. chalepensis* has the highest concentration of the elements calcium, chromium, zinc, copper, and molybdenum in descending order (Table 7).

**Table 7:** Quantitative elemental analysis of *Ruta chalepensis* plant extract by Atomic absorption spectroscopy.

No	Elements	Concentration (ppm)
1	Zinc	55.72
2	Copper	39.78
3	Calcium	308.24
4	Chromium	111.1
5	Molybdenum	8.12

Elemental analysis of the *A. sativum* Aqoues extracts presented in Tables 8 showed that the plant contained major elements like potassium, calcium, magnesium, phosphorous, and aluminum. Quantity minerals, which are potassium, calcium, aluminum, magnesium, and phosphorous, have values that ranged between 21000.8, 400.4, 33.6, 1200.5, and 5000.23 ppm, respectively.

**Table 8:** Quantitative elemental analysis of *Allium sativum* plant extract by FAAS.

No	Elements	Concentration (ppm)
1	Potassium	21000.8
2	Calcium	400.4
3	Aluminum	33.6
4	Magnesium	1200.5
5	Phosphorous	5000.23

The values of the various metals in the Aqoues FAAS extract samples (ppm) are shown in Table 8. The concentration order of the components was as follows: K > P > Mg > Ca > Al. The result of this study is consistent with the study of Yusuf et al. [35], Divya et al. [34], and other authors, in which they showed that the frequency order K > P > Mg > Ca > Zn > Al can improve the healing process of disease and wellbeing, potentially harmful components were not demonstrated [36]. This mineral has numerous functions in human well‐being, such as nerve impulse transmission, muscle contraction, blood pressure regulation, acid‐base balance maintenance, antioxidant activity, and water‐electrolyte balance [37].

On the other hand, in the elemental study of *R. chalepensis*, elements such as zinc, copper, chromium, and molybdenum were determined and their concentration was measured using the comparative strategy of FAAS strategies. The concentration result is shown in Table 7. The analysis of the present studies revealed a wide range of variations in their element concentrations. The concentration of calcium and chromium is high and the remaining components appear in low levels. These minerals play an important role in inflammation and various physiological processes and are crucial for the proper functioning of the immune system. They can influence asthma status [38].

## 4. Conclusion

In the present study, various extracts of *R. chalepensis* and *A. sativum* were investigated for their antibacterial potential and biofilm inhibition test. The antibacterial activity showed significant effects against *E. coli*, S. *aureus* and *S. gallinarum*. The maximum antibacterial activity of *A. sativum* and *R. chalepensis* extract was found against *S. aureus* and the minimum activity against *S. gallinarum*. This suggests the potential role of our medicinal plant as an organic antimicrobial agent. The observed antibacterial efficacy of our raw extract presents a promising alternative for the food industry, potentially replacing chemical preservatives in the production of organic food. In the context of public health, conventional antimicrobial treatments sometimes fail, resulting in prolonged illnesses and increased mortality risks. The antimicrobial agents derived from *A. sativum* exhibit bacteriostatic and bactericidal effects akin to those of currently employed antibiotics, suggesting potential clinical utility in managing specific microbial infections. Consequently, we anticipate that the significant antibacterial properties identified in our investigation will contribute to the mitigation of various diseases prevalent in the studied region.

On the other hand, the present research identified the antibiofilm property of *A. sativum* and *R. chalepensis* extract and found that this plant extract counteracts the proliferation and biofilm formation of *S. aureus*. These results showed that our medicinal plant, known as a traditional Ethiopian spice, is good for oral health and could be considered as a potential novel anticaries agent. Furthermore, as our study shows, it is a simple method to find various functional groups and metals in plants through FT‐IR and FAAS analysis. These functional groups and metals provide us with information about the components present in the plant. This equipment revealed the presence of specific functional groups and metals in *A. sativum* and *R. chalepensis*. Therefore, we hope that the important properties of *A. sativum* and *R. chalepensis* identified in our study will be helpful for drug preparation and antifungal and antibacterial activities.

In conclusion, the present study demonstrates in vitro antibacterial and antibiofilm activity at high concentrations, providing preliminary evidence of biological activity. These findings should be regarded as proof‐of‐concept results, and further investigations using standardized quantitative antimicrobial assays, such as MIC and MBC determination, are required before any potential practical or therapeutic relevance can be assessed.

## Author Contributions

Fitsum Dejene : investigation, methodology, writing—original draft, conceptualization, and formal analysis. Ribka Getu: investigation, methodology, writing—original draft, conceptualization, and formal analysis. Dinka Ejeta: investigation, methodology, writing—original draft, conceptualization, and formal analysis. Yonas Syraji: investigation, writing—original draft, methodology, writing—review and editing, conceptualization, and formal analysis.

## Funding

No funding was received for this manuscript.

## Ethics Statement

This study does not require ethical approval or consent to participate.

## Consent

The authors have nothing to report.

## Conflicts of Interest

The authors declare no conflicts of interest.

## Data Availability

The article contains all the required information.

## References

[1] Wingender J. , Neu T. , and Flemming H. , What Are Bacterial Extracellular Polymeric Substances?, Microbial Extracellular Polymeric Substances, 1999, Springer, 1–19, 10.1007/978-3-642-60147-7_1.

[2] Geethashri A. , Manikandan R. , Ravishankar B. , and Shetty A. , Comparative Evaluation of Biofilm Suppression by Plant Extracts on Oral Pathogenic Bacteria, Journal of Applied Pharmaceutical Science. (2014) 4, no. 3, 20–23, 10.7324/JAPS.2014.40305.

[3] Karatan E. and Watnick P. , Signals, Regulatory Networks, and Materials That Build and Break Bacterial Biofilms, Microbiology and Molecular Biology Reviews. (2009) 73, no. 2, 310–347, 10.1128/mmbr.00041-08, 19487730. 19487730 PMC2698413

[4] McConoughey S. , Howlin R. , Granger J. , Manring M. , Calhoun J. , Shirtliff M. , and Stoodley P. , Biofilms in Periprosthetic Orthopedic Infections, Future Microbiology. (2014) 9, no. 8, 987–1007, 10.2217/fmb.14.64, 25302955. 25302955 PMC4407677

[5] Ribeiro M. , Monteiro F. , and Ferraz M. , Infection of Orthopedic Implants With Emphasis on Bacterial Adhesion Process and Techniques Used in Studying Bacterial-Material Interactions, Biomatter. (2012) 2, no. 4, 176–194, 10.4161/biom.22905, 23507884. 23507884 PMC3568104

[6] Parvizi J. , Pawasarat I. , Azzam K. , Hansen E. N. , Bozic K. J. , and Austin M. S. , Periprosthetic Joint Infection: The Economic Impact of Methicillin Resistant Infections, Journal of Arthroplasty. (2010) 25, no. 3, e42, 10.1016/j.arth.2010.01.050. 20570103

[7] Song X. , Perencevich E. , Campos J. , Short B. , and Singh N. , Clinical and Economic Impact of Methicillin-ResistantStaphylococcus aureusColonization or Infection on Neonates in Intensive Care Units, Infection Control & Hospital Epidemiology. (2010) 31, no. 2, 177–182, 10.1086/649797. 20001732

[8] Agbor G. , Kuate D. , and Oben J. E. , Antioxidants: Case Study in Cameroon, Pakistan Journal of Biological Sciences.(2007) 10, no. 4, 537–544, 19069532. 19069532 10.3923/pjbs.2007.537.544

[9] Odey M. , Iwara I. , Udiba U. , Johnson J. , Inekwe U. , and Asenye M. , Preparation of Plant Extracts From Indigenous Medicinal Plants, International Journal of Science and Technology. (2012) 1, no. 12, 688–692.

[10] Singh D. and Singh V. , Pharmacological Effects of Allium Sativum L. (Garlic), Annual Review of Biomedical Sciences. (2008) 10, 6–26, 10.5016/1806-8774.2008.v10p6.

[11] De Sa R. Z. , Rey A. , Argañaraz E. , and Bindstein E. , Perinatal Toxicology of Ruta chalepensis (Rutaceae) in Mice, Journal of Ethnopharmacology. (2000) 69, no. 2, 93–98, 10.1016/S0378-8741(98)00232-3. 10687865

[12] Abraham G. , Kechero Y. , Andualem D. , and Dingamo T. , Indigenous Legume Fodder Trees and Shrubs With Emphasis on Land Use and Agroecological Zones: Identification, Diversity, and Distribution in Semi-Humid Condition of Southern Ethiopia, Veterinary Medicine and Science. (2022) 8, no. 5, 2126–2137, 10.1002/vms3.858, 35667022. 35667022 PMC9514486

[13] Demissie A. , Chinthapalli B. , Tenaw S. , and Chitra D. , Cultivation of Micro-Algae for Production of Biodiesel: An Optimized Process, Research in Biotechnology. (2016) 7, 68–78, 10.19071/rib.2016.v7.3037.

[14] Ismail R. , Saleh A. , and Ali K. , GC-MS Analysis and Antibacterial Activity of Garlic Extract With Antibiotic, Journal of Medicinal Plants Studies. (2020) 8, no. 1, 26–30.

[15] Andualem B. , Combined Antibacterial Activity of Stingless Bee (Apis mellipodae) Honey and Garlic (Allium sativum) Extracts Against Standard and Clinical Pathogenic Bacteria, Asian Pacific Journal of Tropical Biomedicine. (2013) 3, no. 9, 725–731, 10.1016/s2221-1691(13)60146-x, 23998014. 23998014 PMC3757282

[16] Adetuyi A. and Popoola A. , Extraction and Dyes Ability Potential Studies of the Colourant in Zanthoxylum Zanthoxyloides Plant on Cotton Fabric, Journal of Science Engineering Technology. (2001) 8, no. 2, 3291–3299.

[17] Trease G. and Evans W. , Pharmacognosy, 1989, 11th edition, Brailliar Tiridacanb Macmillian Publishers.

[18] Syraji Y. , Kibebew M. , Techane Y. , and Albene D. , Antimicrobial, Antiradical Activity, and X Ray Fluorescence Spectroscopy Analysis of Aloe otallensis Plant Used in Traditional Medicine in Southern Ethiopia, International Journal of Microbiology. (2024) 2024, no. 1, 1981990, 10.1155/2024/1981990, 39161649. 39161649 PMC11333140

[19] Sarpeleh A. , Sharifi K. , and Sonbolkar A. , Evidence of Antifungal Activity of Wild Rue (Peganum harmala L.) on Phytopathogenic Fungi, Journal of Plant Diseases and Protection.(2009) 116, no. 5, 208–213, 10.1007/BF03356312.

[20] Berhanu A. , Microbial Profile of Tella and the Role of Gesho (Rhamnus prinoides) as Bittering and Antimicrobial Agent in Traditional Tella (Beer) Production, International Food Research Journal. (2014) 21, no. 1, 357–365.

[21] Bobby M. , Wesely E. , and Johnson M. , FT-IR Studies on the Leaves of Albizia lebbeck Benth, International Journal of Pharmacy and Pharmaceutical Sciences. (2012) 4, no. 3, 293–296.

[22] Bedada T. and Abebaw A. , Metallic Nutrients in Enset (Ensete Ventricosum) Corm and Soil Sample From Some West Shoa Zone, Oromia Regional State, Ethiopia, International Journal of Agricultural Science and Food Technology. (2021) 7, no. 1, 73–80, 10.17352/2455-815X.000091.

[23] Alsaid M. , Daud H. , Bejo S. , and Abuseliana A. , Antimicrobial Activities of Some Culinary Spice Extracts Against Streptococcus agalactiae and Its Prophylactic Uses to Prevent Streptococcal Infection in red Hybrid Tilapia (Oreochromis sp.), World Journal of Fish and Marine Sciences. (2010) 2, no. 6, 532–538.

[24] Ohara A. , Saito F. , and Matsuhisa T. , Screening of Antibacterial Activities of Edible Plants Against Streptococcus Mutans, Food Science and Technology Research. (2008) 14, no. 2, 190–193, 10.3136/fstr.14.190.

[25] Marami L. , Dilba G. , Babele D. , Sarba E. , Gizaw A. , Bune W. , Bayu M. D. , Admasu P. , Mekbeb A. , Tadese M. , Abdisa K. , and Bayisa D. , Phytochemical Screening and In-Vitro Evaluation of antibacterial Activities of Echinops amplexicaulis, Ruta chalepensis and Salix subserrata Against Selected Pathogenic Bacterial Strains in West Shewa Zone Ethiopia, Journal of Experimental Pharmacology. (2021) 13, 511–520, 10.2147/JEP.S305936, 34040458. 34040458 PMC8140919

[26] Nurrahman H. and Widyarman A. , Effectiveness of Matricaria chamomilla Essential Oil on Aggregatibacter actinomycetemcomitans and Treponema Denticola Biofilms, Journal of Indonesian Dental Association. (2020) 3, no. 2, 77–82.

[27] Hans V. , Grover H. S. , Deswal H. , and Agarwal P. , Antimicrobial Efficacy of Various Essential Oils at Varying Concentrations Against Periopathogen Porphyromonas Gingivalis, Journal Of Clinical And Diagnostic Research. (2016) 10, no. 9, ZC16–ZC19, 10.7860/jcdr/2016/18956.8435, 27790572. PMC5072072 27790572

[28] Defa K. , Shiferaw G. , and Feleke S. , Total Phenolic Compound, Antioxidant Activity of Cultivated Ethiopian Ruta chalepensis Crude Extract and Its Essential Oils, International Journal of Basic and Applied Sciences. (2017) 6, no. 3, 83–91.

[29] Jaradat N. , Adwan L. , K’aibni S. , Zaid A. , Shtaya M. , Shraim N. , and Assali M. , Variability of Chemical Compositions and Antimicrobial and Antioxidant Activities of Ruta chalepensis Leaf Essential Oils From Three Palestinian Regions, BioMed Research International. (2017) 2017, 2672689, 10.1155/2017/2672689, 29230405. 29230405 PMC5694611

[30] Baser K. , Özek T. , and Beis S. , Constituents of the Essential Oil ofRuta chalepensisL. From Turkey, Journal of Essential Oil Research. (1996) 8, no. 4, 413–414, 10.1080/10412905.1996.9700650.

[31] Bagchi G. , Dwivedi P. , Singh A. , Haider F. , and Naqvi A. , Variations in Essential Oil Constituents at Different Growth Stages ofRuta chalepensison Cultivation at North Indian Plains, Journal of Essential Oil Research. (2003) 15, no. 4, 263–264, 10.1080/10412905.2003.9712137.

[32] Rustaiyan A. , Khossravi M. , Sultani-Lotfabadi F. , Yari M. , Masoudi S. , and Monfared A. , Constituents of the Essential Oil ofRuta chalepensisL. From Iran, Journal of Essential Oil Research. (2002) 14, no. 5, 378–379, 10.1080/10412905.2002.9699892.

[33] Songsungkan J. and Chanthai S. , Determination of Synergic Antioxidant Activity of the Methanol/Ethanol Extract of Allicin in the Presence of Total Phenolics Obtained From the Garlic Capsule Compared With Fresh and Baked Garlic Clove, International Food Research Journal. (2014) 21, no. 6.

[34] Divya B. J. , Suman B. , Venkataswamy M. , and Thyagaraju K. , A Study on Phytochemicals, Functional Groups and Mineral Composition of Allium sativum (Garlic) Cloves, International Journal of Current Pharmaceutical Research. (2017) 9, no. 3, 10.22159/ijcpr.2017.v9i3.18888.

[35] Yusuf A. , Fagbuaro S. , and Fajemilehin S. , Chemical Composition, Phytochemical and Mineral Profile of Garlic (Allium sativum), Journal of Bioscience and Biotechnology Discovery. (2018) 3, no. 5, 105–109, 10.31248/JBBD2018.073.

[36] Karppanen H. , Minerals and Blood Pressure, Annals of Medicine. (1991) 23, no. 3, 299–305, 10.3109/07853899109148064. 1930921

[37] Estrada-Dominguez V. , Sanchez-Chavez E. , de-la-Cruz-Lázaro E. , Marquez-Quiroz C. , and Osorio-Osorio R. , Effect of Zinc Chelate and Sulfate on Mineral Content, Antioxidant Activity and Grain Yield of V igna unguiculata L, Philippine Agricultural Scientist. (2020) 103, no. 1, 47–54, 10.62550/FE27074019.

[38] Gray R. , Duncan A. , Noble D. , Imrie M. , O′Reilly D. , Innes J. , Porteous D. J. , Greening A. P. , and Boyd A. C. , Sputum Trace Metals Are Biomarkers of Inflammatory and Suppurative Lung Disease, Chest. (2010) 137, no. 3, 635–641, 10.1378/chest.09-1047, 19801580. 19801580

